# Supplementary material for: Rescuing ocular development in an anophthalmic pig by blastocyst complementation
Source: EMBO Mol Med. 2018 Nov 16;10(12):e8861. doi: 10.15252/emmm.201808861 (PMC6284517; doi:10.15252/emmm.201808861)
Supplement: Supplementary file 1 — Appendix [file EMMM-10-e8861-s001.pdf]

Rescuing ocular development in an anophthalmic pig by blastocyst complementation

Hongyong Zhang, Jiaojiao Huang, Zechen Li, Guosong Qin, Nan Zhang, Tang Hai, Qianlong Hong, Qiantao Zheng, Ying Zhang, Ruigao Song, Jing Yao, Chunwei Cao, Jianguo Zhao and Qi Zhou

Appendix

Appendix Table S1
Appendix Table S2
Appendix Table S3

Appendix Table S1. Summary of primer sequence information used for PCR and quantitative RT-PCR.

Gene	Primer sequence (5’-3’)	Product size (bp)
MITF	F:TCCCTCTCAAGGTGCTCGTC<br>R: CACCACCACCAGCGATTGTA	471
MITF-B	F:CTCAAGGTGCTCGTCCAGAT<br>R:GGCAGTAACATGTCGTCACC	418
GFP	F:CACAAGTTCAGCGTGTCCG<br>R:CTCGATGCGGTTCACCAG	300
MITF-qPCR	F:CCAGACATGCGCTGGAACAA<br>R:CAAATGCCGATTGGCGTGC	135
KIT	F:TAAGTGAAAGAAGTCAATCTGAG<br>R:GGCAGTCATGTAACTATCACC	152
ZO-1	F:CCCGGATTCTAAGCAGGGTG<br>R:CCCATACCAACTGGTTCTGCT	212
RPE65	F:CACCTGTTTGATGGGCAAGC<br>R:GCCCGTACGTAAGCATCAGT	101
Bestrophin	F:GCTCATGCGCTATGCCAAC<br>R:GATTCGACCCCCAATCCAGG	223
PAX6	F:CAACCTGGCTAGCGAAAAGC<br>R:GCTGGCAGCCATCTTGAGTA	152
PEDF	F:AGAGAGCCTCACCTCTGAGT<br>R:CCTGCACAGACTTCGTGAGTT	122
**human *ZO-1***	F:CAGCCAGCCTGCTAAACCTA<br>R:GCCATCTCTTGCTGCCAAAC	130
**human *RPE65***	F:CATGGAGTCTTTGGGGAGCC<br>R:TTCCAGCAGCAGAGATCCAC	196
**human *Bestrophin***	F:TCAGCACCGCAGTCTACAAG<br>R:TCTGGAGCAGGATAGGGTCC	198
**human *PAX6***	F:CAACCTGGCTAGCGAAAAGC<br>R:GCTGGCAGCCATCTTGAGTA	286
**human *PEDF***	F:TATCACCTTAACCAGCCTTTCATC<br>R:GGGTCCAGAATCTTGCCAATG	82
GAPDH	F:GCAAAGTGGACATTGTCGCCATCA<br>R:AGCTTCCCATTCTCAGCCTTGACT	124

Appendix Table S2. Production efficiency of E44 chimeric fetuses by complementation of the embryos derived from the *MITF*$^{L247S/L247S}$ cell line with the blastomeres derived from the LW cell line.

Host cell line	Donor cell line	No. of embryos	No. of recipients	Pregnant recipients (%)	No. of E44 fetuses	No. of E44 chimeric fetuses (%)
MITF$^{L247S/L247S}$ fibroblast	LW Fibroblast	3754	16	3 (18.75)	4	1 (25%)

Appendix Table S3. Production efficiency of full-term chimeric piglets by complementation of the embryos derived from the *MITF*$^{L247S/L247S}$ cell line with the blastomeres derived from the Bama GFP-labeled cell line.

Host cell line	Donor cell line	No. of embryos	No. of recipients	Pregnant recipients (%)	No. of piglets	No. of chimeric piglets (%)
MITF$^{L247S/L247S}$ fibroblast	GFP-labeled Fibroblast	1671	7	3 (42.86)	7	1 (14.29%)
