# Supplementary material for: Rescuing ocular development in an anophthalmic pig by blastocyst complementation
Source: EMBO Mol Med. 2018 Nov 16;10(12):e8861. doi: 10.15252/emmm.201808861 (PMC6284517; doi:10.15252/emmm.201808861)
Supplement: Supplementary file 2 — Expanded View Figures PDF [file EMMM-10-e8861-s002.pdf]

Expanded View Figures

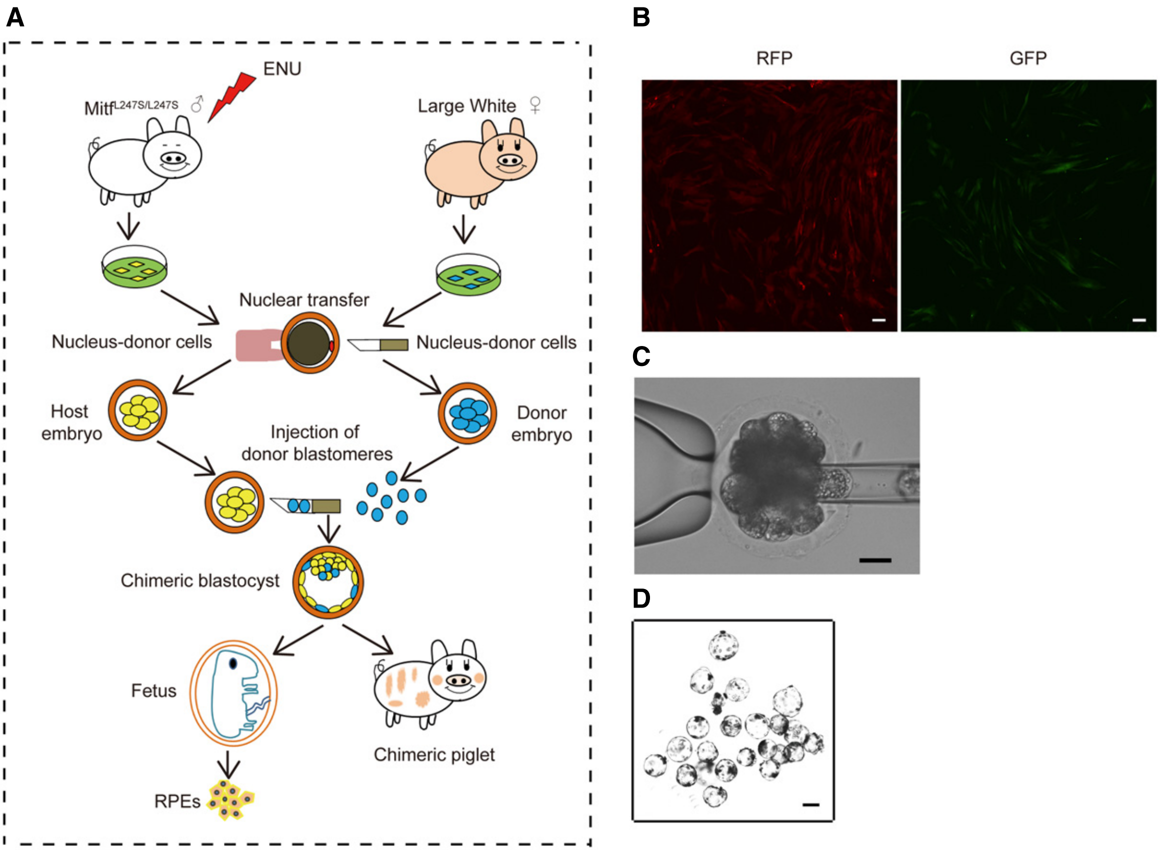

Figure EV1. Generation of GFP- and RFP-labeled PEFs and chimeric porcine blastocysts.

A Schematic procedures for the generation of chimeric fetuses and pigs.

B The PEFs were confirmed by expression of *GFP* and *RFP*, which were then used for SCNT. Scale bars, 100 μm.

C Complementation of cloned host embryos, derived from the Bama RFP-labeled PEFs, with injection of donor blastomeres, derived from Bama GFP-labeled PEFs. Scale bar, 100 μm.

D The chimeric blastocysts. Scale bar, 100 μm.

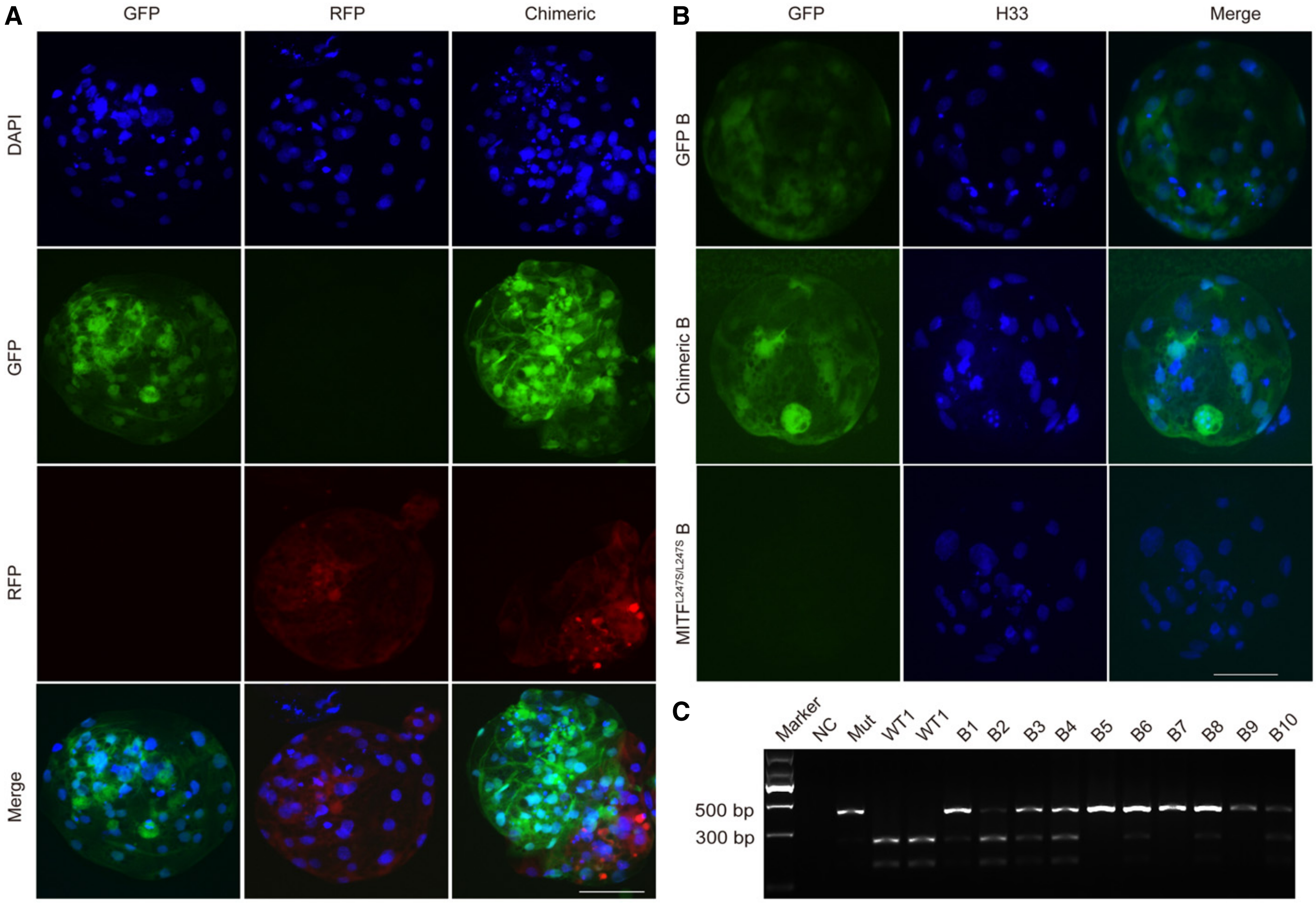

Figure EV2. The chimeric contributions *in vitro* were detected by immunofluorescence and RFLP analysis simultaneously.

A Representative immunofluorescence images show the expression of *RFP* and *GFP* simultaneously in chimeric embryos. GFP-labeled and RFP-labeled blastocysts are shown as negative controls. Scale bar, 50 μm.

B Representative immunofluorescence images showed the expression of *GFP* in the chimeric blastocyst. Scale bar, 50 μm.

C Genotyping for porcine blastocysts derived from injected embryos. Porcine *MITF*-specific primers were used for the detection of chimeric contribution. Restriction enzymes DraI was used for digestion of PCR products. NC, negative control with no genomic DNA loaded. Mut, Bama *MITF*$^{L247S/L247S}$ blastocyst DNA loaded. WT, Bama wild-type blastocyst DNA loaded. B1-B10, collected the injected blastocysts DNA loaded.

 © 2018 The Authors

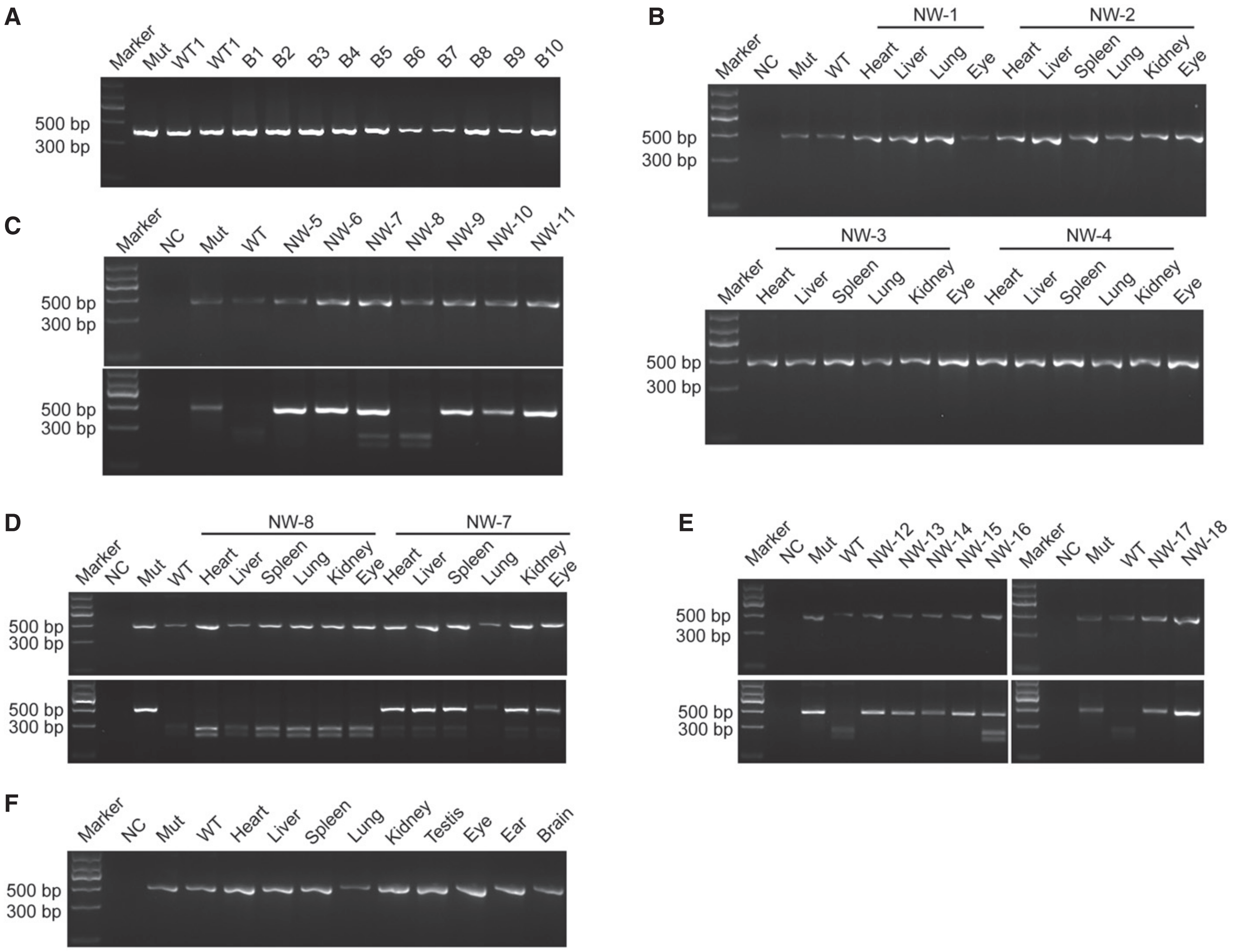

Figure EV3. Genotyping for the chimeric contributions in fetuses and full-term chimeric pig.

A PCR amplification of *MITF* in the blastocysts. Mut, Bama *MITF*$^{L247S/L247S}$ blastocyst DNA loaded. WT1-WT2, DNA of Bama WT number 1 and 2 blastocysts loaded. B1-B10, DNA of the injected blastocysts from number 1 to number 10 loaded.

B PCR amplification of *MITF* in the E44 fetuses. NC, negative control with no genomic DNA loaded. Mut, *MITF*$^{L247S/L247S}$ genomic DNA loaded. WT, LW genomic DNA loaded.

C PCR amplification of *MITF* and PCR-RFLP analysis of *MITF* in E60 fetuses. NC, negative control with no genomic DNA loaded. Mut, *MITF*$^{L247S/L247S}$ genomic DNA loaded. WT, Bama genomic DNA loaded. NW-5-NW-11, fetuses at E60.

D PCR amplification of *MITF* and digestion of the PCR products in the multiple organs of the NW-7 and NW-8 fetuses. NC, negative control with no genomic DNA loaded. Mut, *MITF*$^{L247S/L247S}$ genomic DNA loaded. WT, Bama genomic DNA loaded.

E PCR amplification of *MITF* and PCR-RFLP analysis of *MITF* in the full-term chimeric pig. NC, negative control with no genomic DNA loaded. Mut, *MITF*$^{L247S/L247S}$ genomic DNA loaded. WT, Bama GFP-labeled genomic DNA loaded.

F PCR amplification of *MITF* in multiple organs of the full-term chimeric pig. NC, negative control with no genomic DNA loaded. Mut, *MITF*$^{L247S/L247S}$ genomic DNA loaded. WT, Bama GFP-labeled genomic DNA loaded.

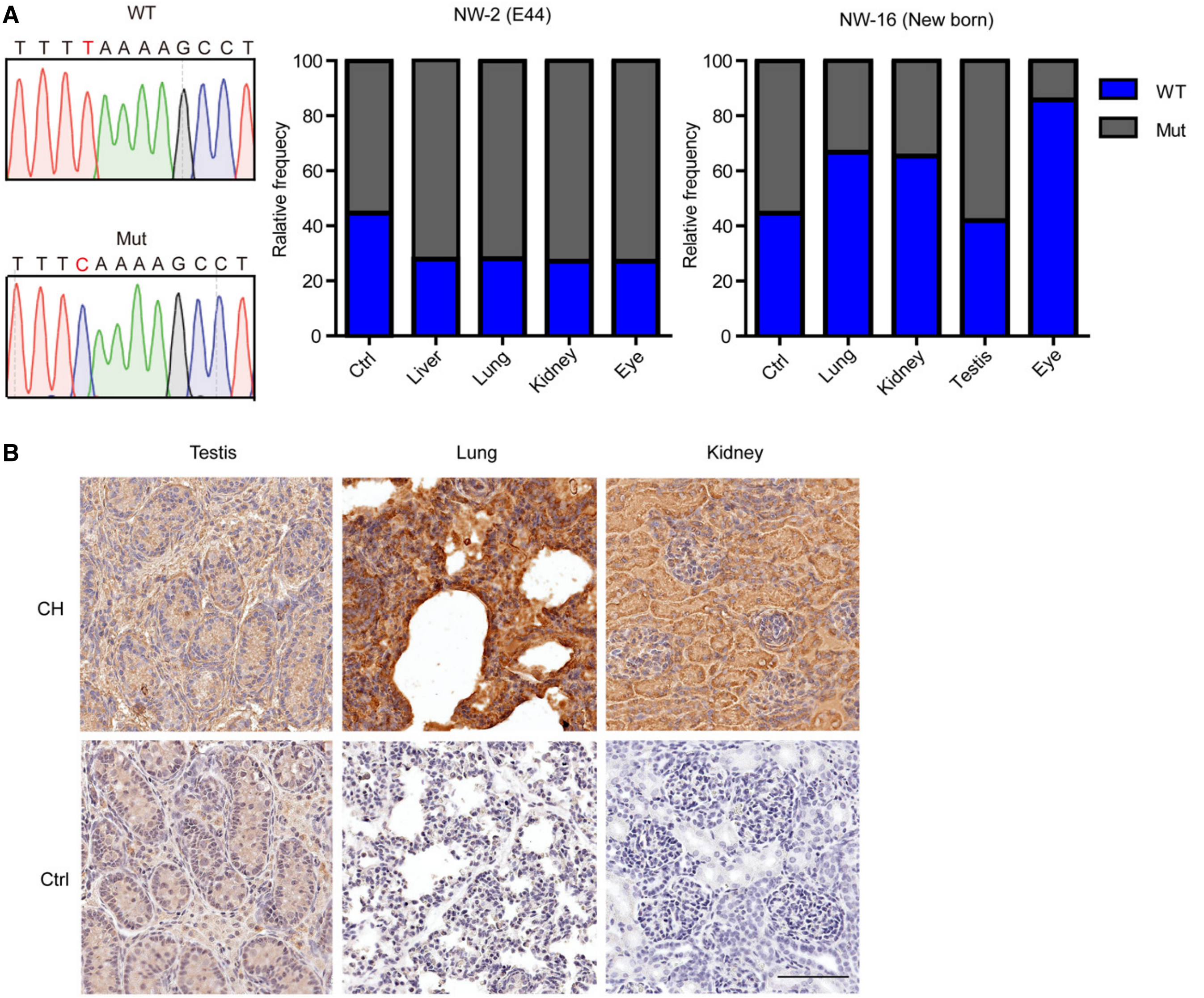

Figure EV4. Identification of the chimeric contributions in tissues from E44 fetus and piglet NW-16.

A Next-generation sequencing analysis of the origin of *MITF* in multiple tissues of E44 fetus and piglet NW-16. The PCR product of *MITF* $^{+/L247S}$ was as control. WT, *MITF* $^{+/+}$ allele. Mut, *MITF* $^{L247S/L247S}$ allele.

B The chimeric contributions were determined in the multiple tissues of the chimeric pig by immunohistochemistry analysis of *GFP*. Scale bar, 90 μm.

 © 2018 The Authors
