# Supplementary figures and images for: Rescuing ocular development in an anophthalmic pig by blastocyst complementation
Source: EMBO Mol Med. 2018 Nov 16;10(12):e8861. doi: 10.15252/emmm.201808861 (PMC6284517; doi:10.15252/emmm.201808861)

Figure 1B

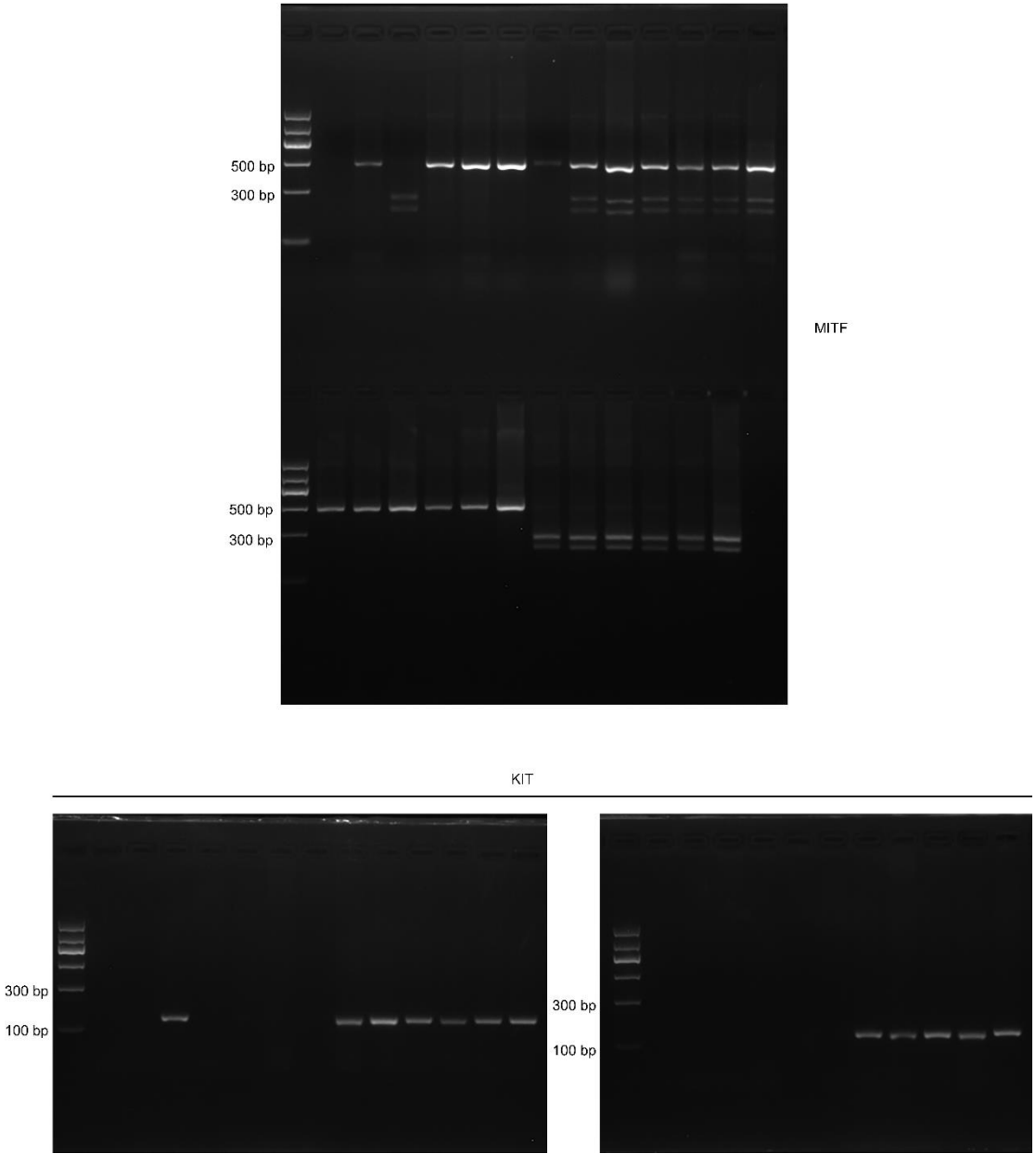

Supplement: Supplementary file 4 — Source Data for Figure 1 [file EMMM-10-e8861-s003.pdf]

Figure 2B

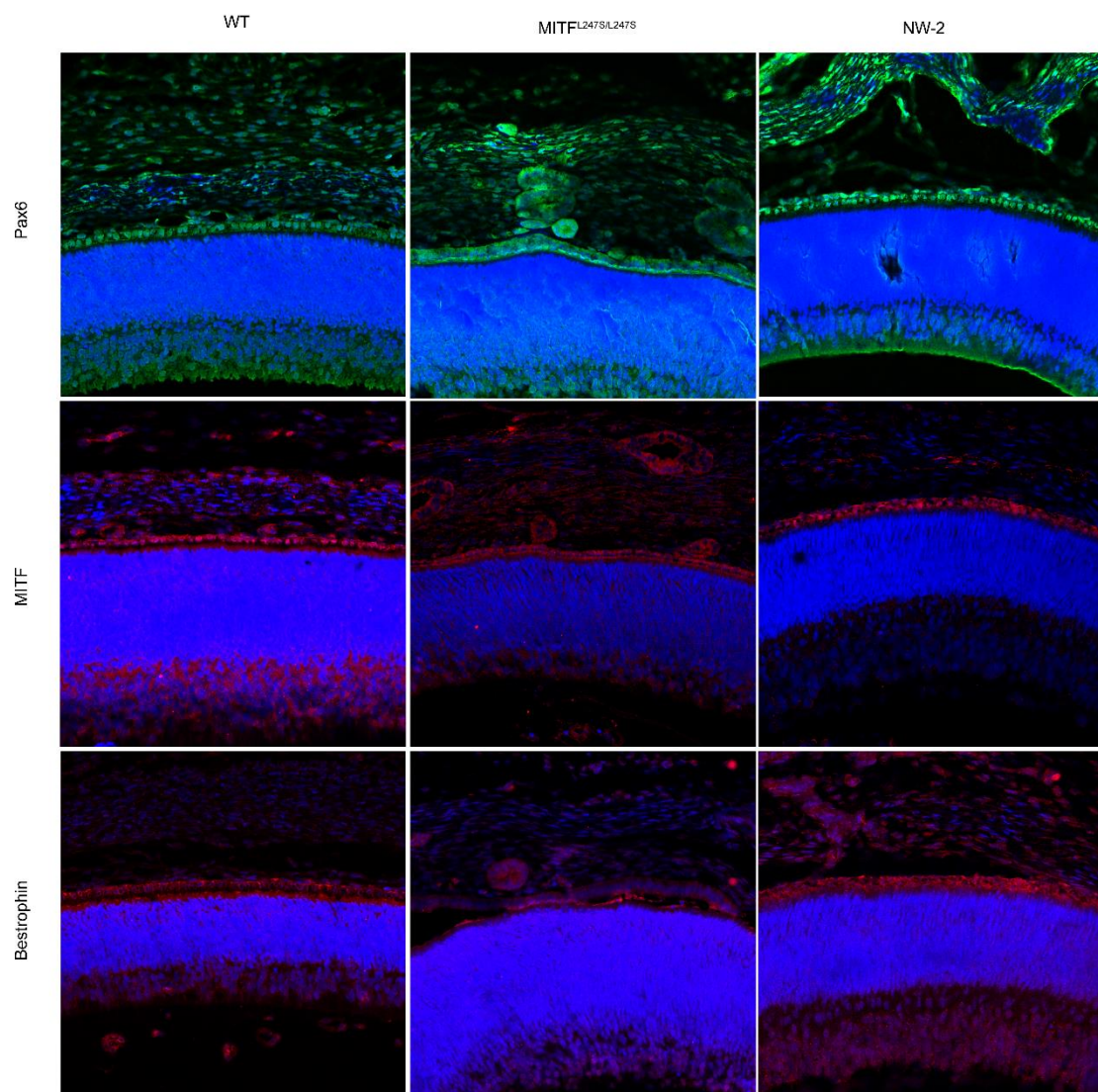

Supplement: Supplementary file 5 — Source Data for Figure 2 [file EMMM-10-e8861-s004.pdf]

Figure 3

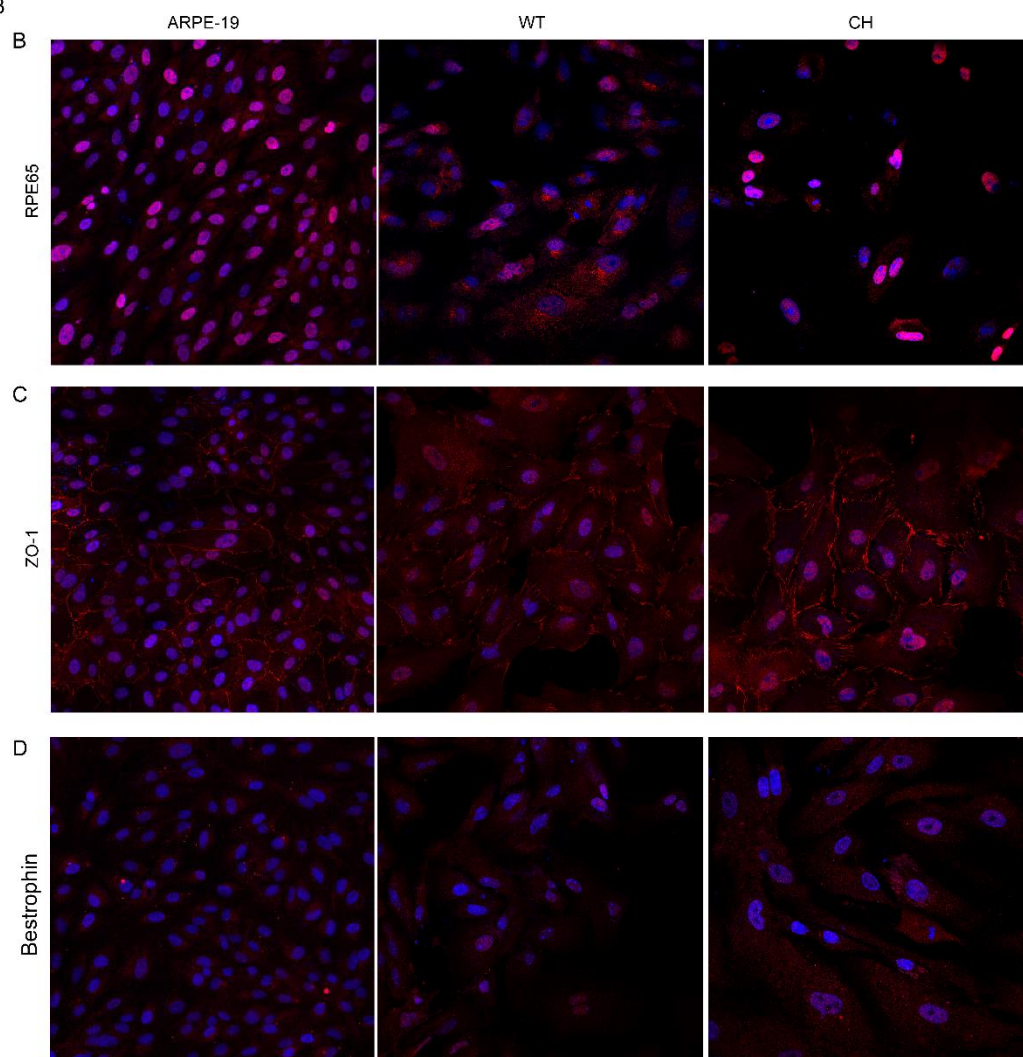

Figure 3

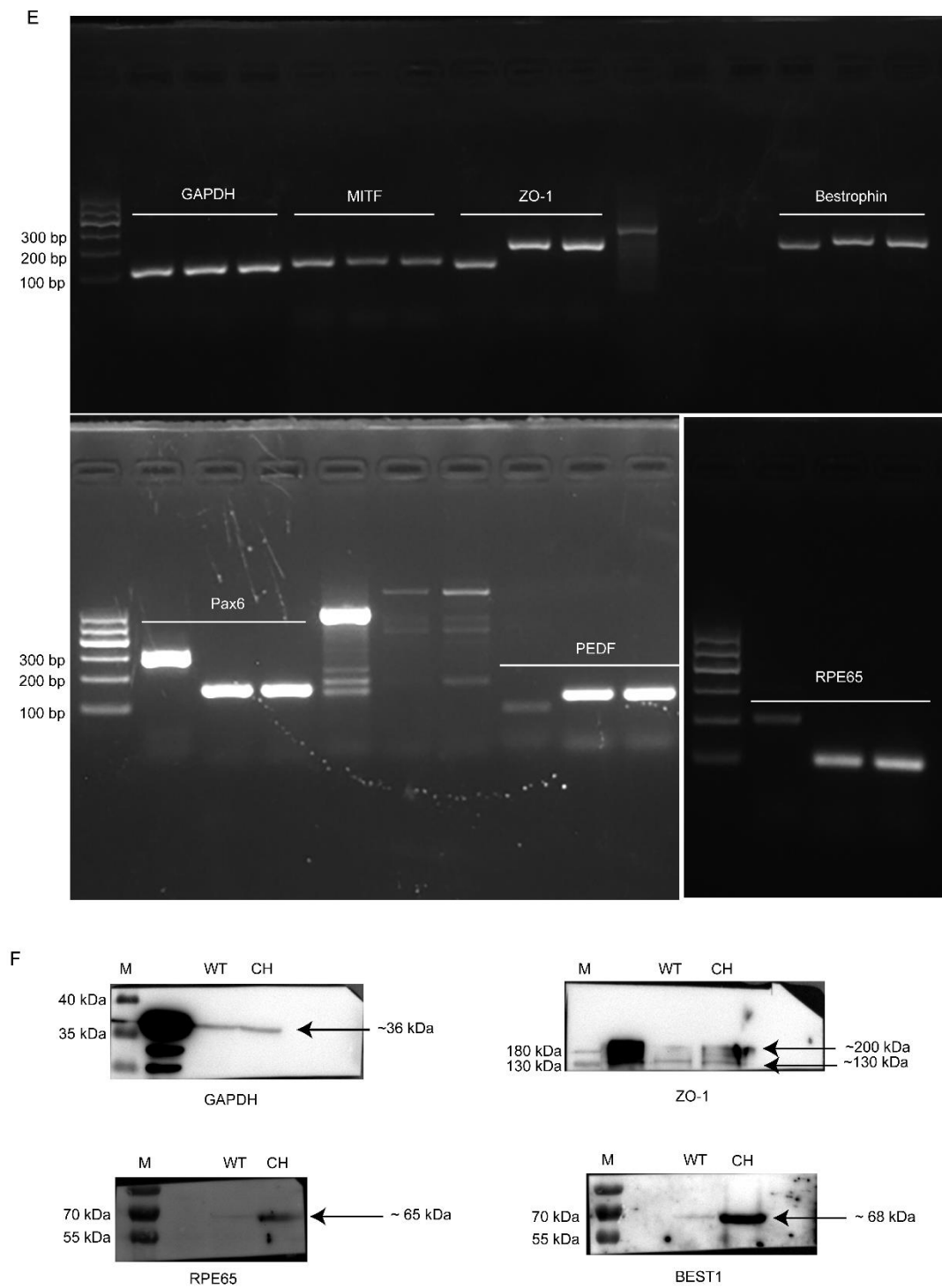

Supplement: Supplementary file 6 — Source Data for Figure 3 [file EMMM-10-e8861-s005.pdf]

Figure 4B

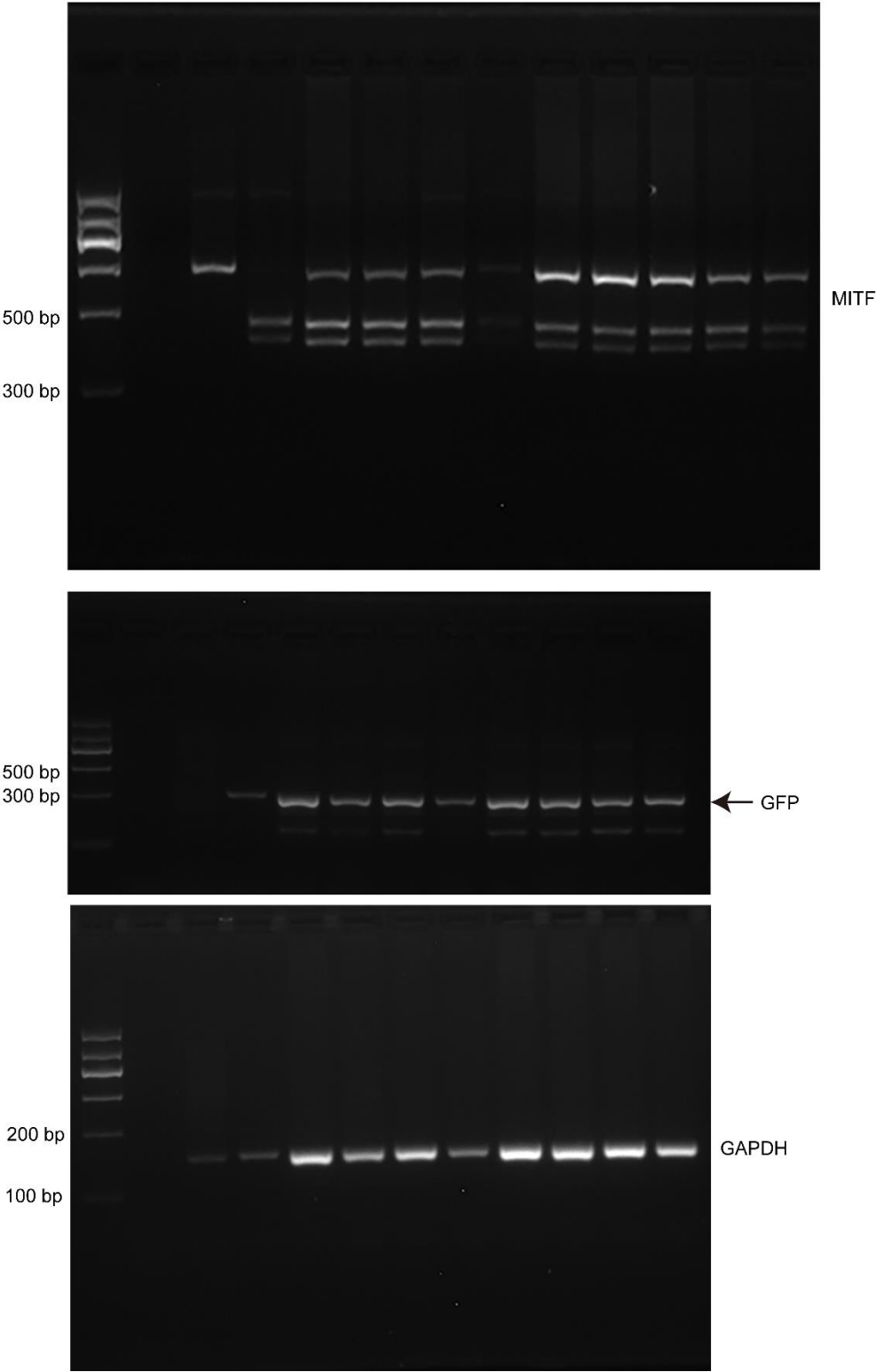

Supplement: Supplementary file 7 — Source Data for Figure 4 [file EMMM-10-e8861-s006.pdf]

Figure 5

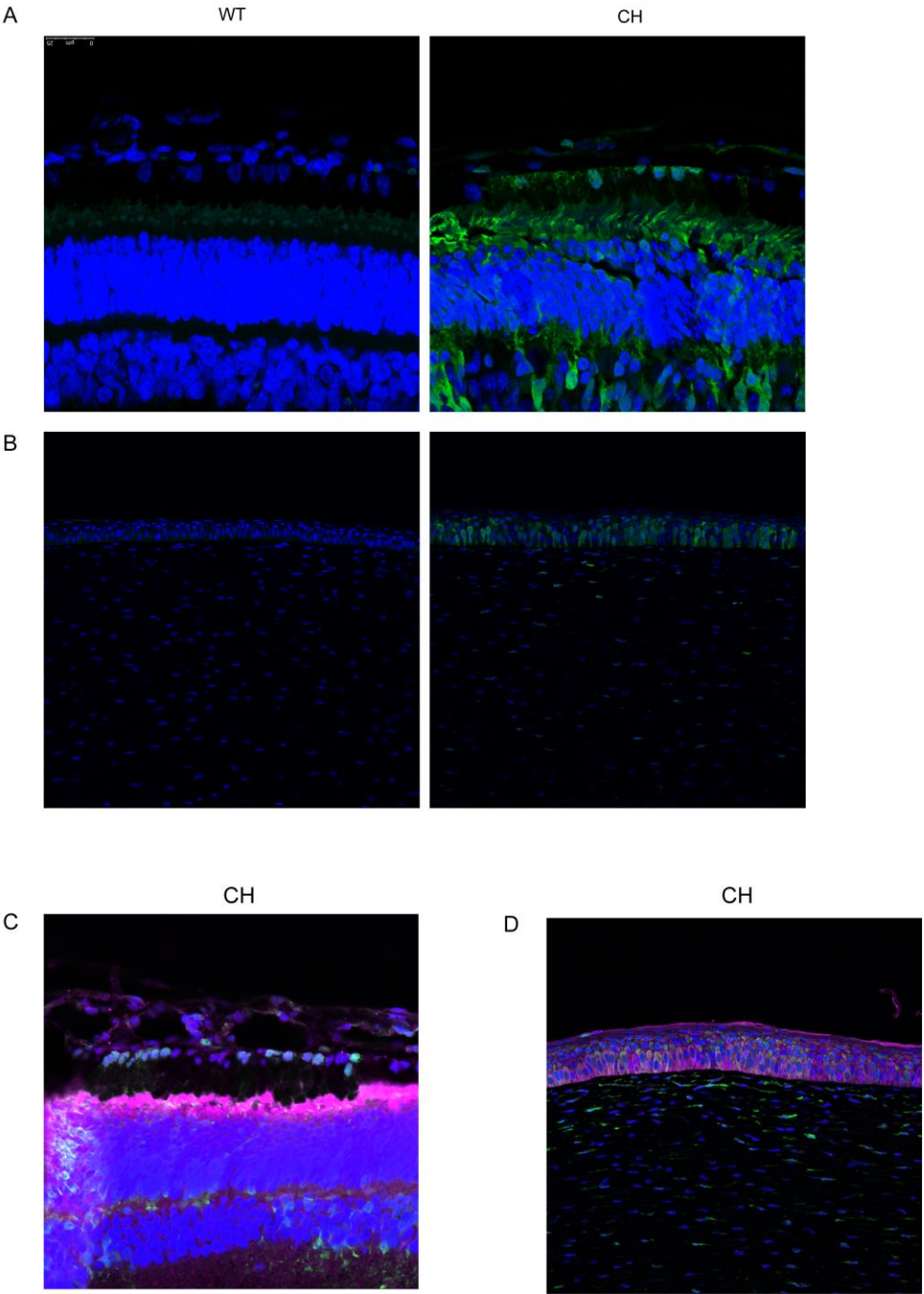

Supplement: Supplementary file 8 — Source Data for Figure 5 [file EMMM-10-e8861-s007.pdf]
